# Whole genome methylation combined with RNA-seq reveals the protective effects of Gualou-Xiebai herb pair in foam cells through DNA methylation mediated PI3K-AKT signaling pathway

**DOI:** 10.3389/fimmu.2023.1054014

**Published:** 2023-02-22

**Authors:** Zijun Jia, Jun Mei, Yan Zhang, Ya Wang, Hongqin Wang, Anlu Wang, Fengqin Xu, Qingbing Zhou

**Affiliations:** ^1^ Xiyuan Clinical Medical College, Beijing University of Chinese Medicine, Beijing, China; ^2^ Institute of Geriatric Medicine, Xiyuan Hospital, China Academy of Chinese Medical Sciences, Beijing, China; ^3^ Cardiovascular Diseases Center, Xiyuan Hospital, China Academy of Chinese Medical Sciences, Beijing, China

**Keywords:** Gualou-Xiebai herb pair, DNA methylation, transcriptome, atherosclerosis, foam cells

## Abstract

DNA methylation, including aberrant hypomethylation and hypermethylation, plays a significant role in atherosclerosis (AS); therefore, targeting the unbalanced methylation in AS is a potential treatment strategy. Gualou-xiebai herb pair (GXHP), a classic herb combination, have been used for the treatment of atherosclerotic-associated diseases in traditional Chinese medicine. However, the effects and underlying mechanism of GXHP on AS remain nebulous. In this study, the CCK-8 method was applied to determine the non-toxic treatment concentrations for GXHP. The formation of foam cells played a critical role in AS, so the foam cells model was established after RAW264.7 cells were treated with ox-LDL. The contents of total cholesterol (TC) and free cholesterol (FC) were determined by Gas chromatography-mass spectrometry (GC-MS). Enzyme-linked immunosorbent assay (ELISA) was used to check the expressions of inflammatory factors including IL-1β, TNF-α, and VCAM-1. Methyl-capture sequencing (MC-seq) and RNA-seq were applied to observe the changes in genome-wide DNA methylation and gene expression, respectively. Kyoto Encyclopedia of Genes and Genomes (KEGG) were performed to analyze differentially methylated genes (DMGs) and differentially expressed genes (DEGs). The targeted signaling pathway was selected and verified using western blotting (WB). The results showed that the lipids and inflammatory factors in foam cells significantly increased. GXHP significantly reduced the expression of TC, FC, and inflammatory factors. MC-seq and RNA-seq showed that GXHP not only corrected the aberrant DNA hypermethylation, but also DNA hypomethylation, thus restored the aberrant DEGs in foam cells induced by ox-LDL. GXHP treatment may target the PI3K-Akt signaling pathway. GXHP reduced the protein levels of phosphorylated(p)-PI3K and p-AKT in foam cells. Our data suggest that treatment with GXHP showed protective effects against AS through the inhibition of DNA methylation mediated PI3K-AKT signaling pathway, suggesting GXHP as a novel methylation-based agent.

## 1 Introduction

Atherosclerosis (AS) is a complex multifactorial disease (1). The risk factors for AS include high low-density lipoprotein (LDL) levels, inflammation, obesity, disturbed sleep, and hyperglycemia (2). Cardiovascular diseases (CVD), in which the pathological basis is AS, are the leading cause of mortality worldwide. It is expected that by 2035, 130 million adults (45.1%) in the United States will suffer from CVDs. Moreover, the mortality rate of CVDs is higher in developing countries due to racial differences, limited access to medical care, and economic development (3). Many patients with AS become incapacitated and may lose their limbs, which increases the economic burden of social public health; thus, the discovery of novel drugs for treating AS diseases is crucial (4, 5).

Recent studies have focused on the relationship between AS and DNA methylation. DNA methylation refers to the process in which “C” bases receive methyl groups from S-adenosine methionine to form 5-methyl cytosine without changing the DNA sequence. The main biological function of DNA methylation in promoter regions is to influence the mRNA expression of genes, thereby up- or downregulating the expression (6). Increasing evidence has shown that DNA methylation plays a key role in AS, while several aberrantly methylated genes exist in AS that are associated with reverse cholesterol transport, inflammatory response, foam cell formation, endothelial cell dysfunction, and abnormal proliferation of vascular smooth muscle cells (7). For example, Kruppel-like factor 2 (KLF2) has an important anti-inflammatory effect. The aberrant hypermethylation of KLF2 has been identified in AS, which decreases mRNA expression, increases inflammation, and indices endothelial dysfunction in vascular endothelial cells (8). However, aberrant hypomethylation also contributes to AS development. Indeed, Yang reported that lectin-like oxidized-LDL (ox-LDL) receptor-1 (LOX-1), a unique receptor involved in the uptake of ox-LDL, is aberrantly hypomethylated in blood vessels from ApoE^-/-^ mice (9). Einari et al. identified 3,997 abnormal hypomethylated sites and 782 abnormal hypermethylated sites in femoral AS plaques from 22 cases compared with 9 normal cases (10). Moreover, gene expression analysis has revealed that the hypomethylation of genes in promoter regions correlates with increased levels of mRNA expression (10). Since DNA methylation is reversible, targeting the aberrant methylation in AS has attracted attention (11, 12).

Gualou-Xiebai herb pair (GXHP), a classic herb combination, originates from *ShangHanZaBingLun*, written by Zhong-jing Zhang during the Eastern Han Dynasty. *Trichosanthes kirilowii Maxim* (Gua lou) and *Allium macrostemon* (Xie bai) have been used for the treatment of AS-associated diseases in traditional Chinese medicine (TCM) (13). Recently, Zhou et al. (14) found that a GXHP decoction could inhibit inflammatory cytokines in the treatment of patients with CVD. However, the treatment mechanism for GXHP remains unclear.

In this study, a multi-omics approach was applied to clarify the molecular mechanism underlying the therapeutic effects of GXHP on AS. First, a foam cell model was constructed with RAW264.7 cells treated with ox-LDL, which was used to observe the anti-AS effects of GXHP. Furthermore, we elucidated the mechanism of GXHP granules *via* methyl-capture sequencing (MC-seq) and RNA-seq. Kyoto Encyclopedia of Genes and Genomes (KEGG) analyses were used to assess the differentially methylated genes (DMGs) and differentially expressed genes (DEGs) among distinct groups. Finally, western blotting was performed to elucidate the effects of GXHP on the PI3K-Akt signaling pathway in foam cells, which were selected according to the integrated MC-seq analysis combined with RNA-seq.

## 2 Materials and methods

### 2.1 Main herbs and reagents

The GXHP granules, comprising Gua lou and Xie bai, were supplied by Sanjiu Medical & Pharmaceutical (Shenzhen, China) at a ratio of 1:1. High glucose Dulbecco’s Modified Eagle Medium (DMEM) and fetal bovine serum (FBS) were supplied by Gibco (New York, USA). Cell viability was determined by a Cell Counting Kit-8 (Dojindo Molecular Technologies, Kumamoto, Japan). Human ox-LDL was obtained from Yiyuan Biotechnology (Guangzhou, China). The GXHP granules were dissolved in DMEM supplemented with 10% inactivated FBS for cell treatment.

### 2.2 CCK-8 cell viability assay

RAW264.7 cells were cultured in high glucose DMEM supplemented with 10% inactivated fetal bovine serum at 37°C. The cells were seeded at a density of 1× 10^4^ cells per well in 96-well plates. Serially diluted concentrations of GXHP were added to the wells and cultured with the cells for 48 h. Then, 10 µL of CCK-8 reagent was added into each well and the absorbance value of each sample was determined with a microplate reader (Biotek, Shanghai, China) at 450 nm. We repeated the experiments thrice and calculated the cell viability of each group.

### 2.3 Establishment of a foam cell model and grouping

As described in the previous study (15), RAW264.7 cells were treated with ox-LDL at 80 μg/mL for 48 h. Oil Red O staining was performed to observe the lipid droplets in the cells. In this study, there were three treatment groups: control group (RAW264.7 cells only), model group (RAW264.7 cells treated with ox-LDL), and GXHP group (RAW264.7 cells treated with ox-LDL and GXHP at 1.8 g/L). The cells in the three groups were collected for MC-seq and RNA-seq.

### 2.4 Measurement of lipids and inflammatory factors

The levels of total cholesterol (TC) and free cholesterol (FC) in cell samples from different groups were determined using gas chromatography-mass spectrometry (GC-MS). The cell samples were loaded onto a GC-MS-TQ8040 NX mass spectrograph (SHIMADZU, Origin, Japan) with an RTX-5MS column. The conditions included an inlet temperature of 280°C and a ramp-up procedure that began at 150°C for 1 min, increased to 300°C at 40°C/min, and maintained for 7 min. The supernatant was collected from different groups. Then the levels of vascular cell adhesion molecule-1 (VCAM-1),tumor necrosis factor (TNF)-α, and interleukin-1β (IL-1β) were determined using an enzyme-linked immunosorbent assay (ELISA) (BioTek, Winooski, VT, USA).

### 2.5 Agilent methyl-capture sequencing (MC-seq)

The cells were cultured in a six-well plate with 5×10^5^ cells per well for 48h.And the total DNA were extracted from the three groups using a DNeasy Blood Tissue Kit (250) All-Prep DNA Mini Kit (Qiagen, Valencia, CA, USA). Agilent MC-seq was applied to assess the methylation status in the nine cell samples (Santa Clara, CA, USA). Briefly, 1.5 μg DNA per sample was needed and the DNA samples library was constructed. The concentration and size of the constructed library were measured with the Qubit^®^ 2.0 fluorometer and Agilent 2100 Bioanalyzer, respectively. Following bisulfite treatment and amplification by polymerase chain reaction (PCR), the DNA samples were then sequenced on Illumina NovaSeq6000 (Illumina, San Diego, CA, USA). The quality of sequence was assessed using the Fastp v0.20.0 Software (https://github.com/OpenGene/fastp). Finally, the eligible differential methylation sites (DMSs) were identified and annotated using the Dispersion Shrinkage and the ClusterProfiler package.

### 2.6 RNA-seq

The total RNA content was extracted from cells using the Total RNA Extraction Kit (Qiagen, Valencia, CA, USA). The concentration and quality of extracted RNA were assessed using a NanoDrop spectrophotometer and an Agilent 2100 Bioanalyzer, respectively. Following purification using RNA Clean XP Kit (Beckman Coulter, Inc. Kraemer Boulevard Brea, CA, USA), 1.5 μg RNA per sample was subjected to RNA-seq. Briefly, the RNA library was constructed using an Illumina TruSeq RNA preparation kit (Illumina, San Diego, CA, USA) according to the manufacturer’s instructions. The RNA samples were then sequenced on the Illumina NovaSeq6000 (Illumina, San Diego, CA, USA). Finally, the eligible differentially expressed genes(DEGs) were identified and annotated with the use of the DSS (Shrinkage for Sequencing Data) tool in the Bioconductor Package.

### 2.7 Bioinformatics analyses

KEGG pathway enrichment analyses were performed using the DAVID Bioinformatics Resources online data platform (https://david.ncifcrf.gov/home.jsp). The related pathways and functions were selected based on the criterion *P* < 0.05; we selected the top 20.

### 2.8 Western blotting

Cells were divided into the following five groups for western blotting: control group (untreated RAW 264.7 cells), model group (RAW 264.7 cells treated with ox-LDL), and three treatment groups (RAW 264.7 cells treated with ox-LDL and 0.2, 0.6, and 1.8 g/L of GXHP, respectively). Cells were collected, and a protein extraction kit (Gene pool, Beijing, China) was used to extract proteins according to the manufacturer’s protocol. The protein concentration was determined using a BCA protein assay (Multi Sciences, Hangzhou, China). Protein separation using 12% SDS-PAGE gel was then performed, and the protein samples were transferred to a PVDF membrane. After blocking, the PVDF membrane was incubated with specific primary antibodies (Cell Signaling Technology, Danvers, MA, USA), such as PI3 kinase p85 antibody (dilution ratio was 1:500), phospho-PI3 kinase p85 antibody (dilution ratio was 1:1,000), AKT1 antibody (dilution ratio was 1:1500), and phospho-AKT1 antibody (dilution ratio was 1:500), overnight at 4°C. This was followed by 1 hour incubation at room temperature with horseradish peroxidase conjugated goat anti-rabbit IgG (Abcam, Cambridge, MA, USA)(dilution ratio was 1:5000).Antigen–antibody binding was detected using enhanced chemiluminescence reagents (ThermoFisher Scientific, Waltham, MA, USA). Quantification of each protein was determined using Quantity One v.4.6.2.

### 2.9 Statistical analysis

Differences in the levels of cholesterol, inflammatory factors, and protein expression among different groups were assessed using one-way ANOVA coupled with Tukey’s multiple comparison test. *P* < 0.05 was considered statistically significant. Differential methylation sites (DMSs) were identified and annotated by the dispersion shrinkage for sequencing data tool in the Bioconductor package, and the thresholds were set as *P* < 0.05 and a cutoff of 0.1 for DNA methylation changes. DEGs were defined by a false discovery rate (*q*) < 0.05 and a log_2_ fold change threshold of > 0.5 or < -0.5. Correlation analysis was performed using Pearson’s correlation coefficient.

## 3 Results

### 3.1 Effect of GXHP on cell viability of RAW264.7 cells

**Figure 1:**
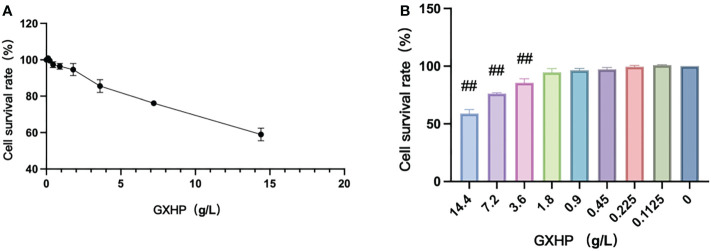
Effects of GXHP on cell viability of RAW264.7 cells. **(A)** Dose–response curve for GXHP. **(B)** Comparison of cell survival rate (%) among different groups following treatment with GXHP at different concentrations ranged from 0 to 14.4 g/L for 48 h. The error bars indicate mean ± SEM. Results were from three independent experiments. ##*P* < 0.01, compared with control group.

As illustrated in **Figure 1A**, the cell viability of RAW264.7 cells was assessed using a CCK-8 assay. GXHP had dose-dependent effects on cell viability. **Figure 1B** shows that treatment with GXHP at a concentration of 3.6 mg/mL could significantly reduce the viability of RAW264.7 cells. Thus, a concentration of ≤ 1.8 g/L for GXHP was selected as the treatment concentration for subsequent experiments.

### 3.2 GXHP reduced the levels of TC, FC, and inflammatory factors in foam cells

**Figure 2:**
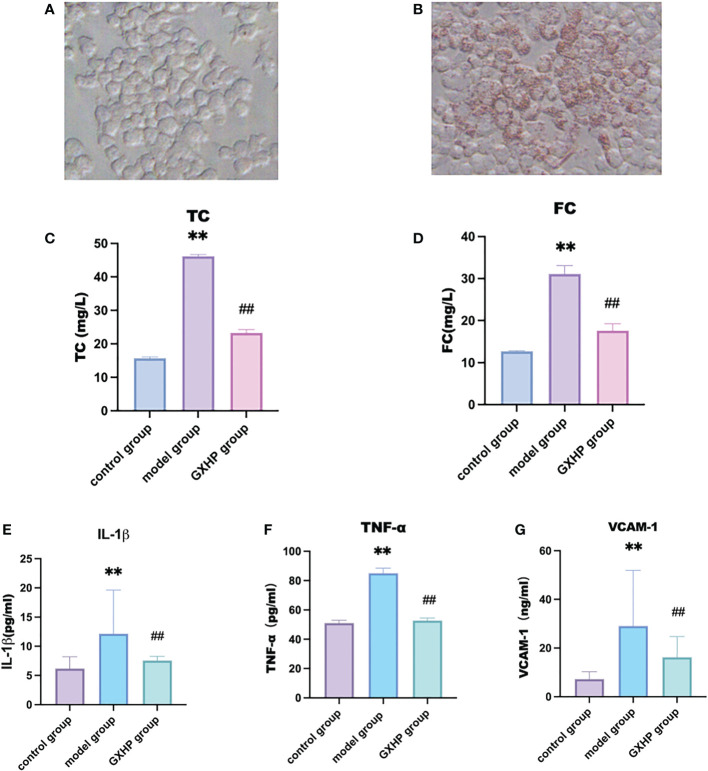
Effects of GXHP on expressions of inflammatory factors in foam cells. Cells were from control group **(A)** and foam cell model group **(B)** after oil red O staining (200×). Levels of **(C)** TC, **(D)** FC, **(E)** IL-1β, **(F)** TNF-α, and **(G)** VCAM-1. Each bar represents the mean ± SD. Results were from five independent experiments. **P < 0.01, compared with the control group; ##*P* < 0.01, compared with the model group.

Compared with the control group, Oil Red O staining showed that orange-red lipid droplets assembled in foam cells following treatment of RAW264.7 cells with ox-LDL at 80 μg/L (**Figures 2A, B**). Compared with the control group, ox-LDL treatment could significantly increase the levels of TC and FC in the model group (*P* < 0.01). Compared with the model group, TC and FC expression in the GXHP group decreased significantly (*P* < 0.01) (**Figures 2C, D**). The expression levels of IL-1β, TNF-α, and VCAM-1 in the model group were significantly higher than those in the control group (all *P* < 0.01). Compared with the model group, GXHP could downregulate the expressions of IL-1β, TNF-α, and VCAM-1 (*P* < 0.01) (**Figures 2E** **–G**).

### 3.3 GXHP reversed DNA methylation changes in RAW264.7 cells treated with ox-LDL

**Figure 3:**
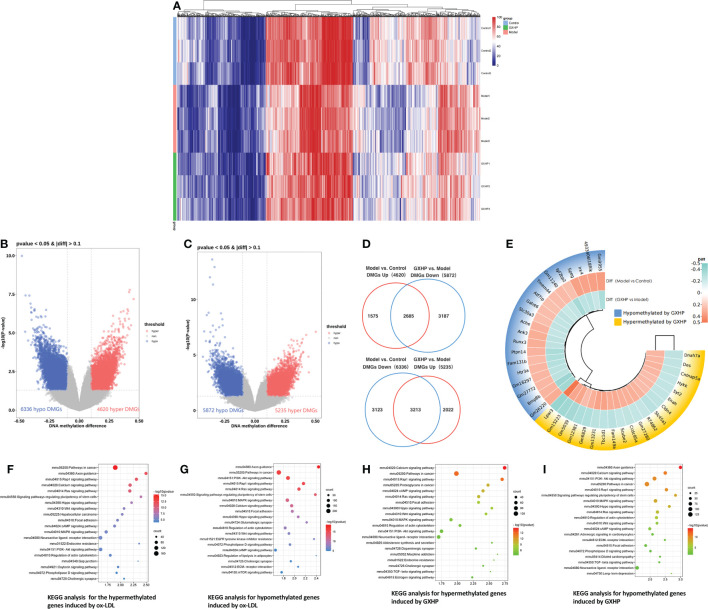
Effects of GXHP on DNA methylation in foam cells. **(A)** Heatmap showing the top 1,000 differentially methylated genes (DMGs) among GXHP, model, and control groups. **(B)** Volcano plot showing the DMGs between model and control groups. **(C)** Volcano plot showing the hypermethylated and hypomethylated genes DMGs between GXHP and model groups. **(D)** Venn diagram showing the differentially hypermethylated and hypomethylated genes induced by GXHP and ox-LDL. **(E)** Circle heatmap showing the top 20 hypermethylated and hypomethylated genes in the model group versus the control group. **(F–I)** Top 20 KEGG pathways for the differentially hypermethylated and hypomethylated genes induced by ox-LDL or GXHP.

In this study, MC-seq was used to determine whether GXHP treatment could alter the status of genome-wide DNA methylation in foam cells. The heatmap intuitively displayed the differential methylation in the nine samples from the three groups (**Figure 3A**). Compared with the control group, volcano plots showed 10,956 DMGs in the model group corresponding to 6,336 hypomethylated and 4,620 hypermethylated genes (**Figure 3B**). We also identified 11,107 DMGs between the GXHP and model groups, including 5,235 hypermethylated genes and 5,872 hypomethylated genes (**Figure 3C**).

Of the DMGs regulated by ox-LDL and GXHP, 5,898 DMGs change DNA methylation levels in opposite directions. Specifically, 2,685 ox-LDL-induced hypermethylated genes were hypomethylated by GXHP treatment. Furthermore, 3,213 DMGs hypomethylated by ox-LDL were hypermethylated by GXHP treatment (**Figure 3D**). The circle heatmap shows the top 20 hypermethylated and hypomethylated genes induced by GXHP treatment, which included Bmp8b, Fam131b, Igf2bp2, Lpar3, Tbc1d1, and Ccdc85a (**Figure 3E**). These data suggest that GXHP treatment can reverse the DNA methylation changes induced by ox-LDL in foam cells.

Additionally, KEGG pathway analysis was performed to reveal the possible biological functions related to DMSs. The 6,336 hypomethylated and 4,620 hypermethylated genes induced by ox-LDL were involved in the Rap1, Hippo, Wnt, MAPK, and PI3K-Akt signaling pathways (**Figures 3F, G**). The 5,235 hypermethylated and 5,872 hypomethylated genes induced by GXHP were related to the PI3K-Akt, Rap1, MAPK, and Hippo signaling pathways (**Figures 3H, I**).

### 3.4 GXHP restored gene expression changes in RAW264.7 cells treated by ox-LDL

**Figure 4:**
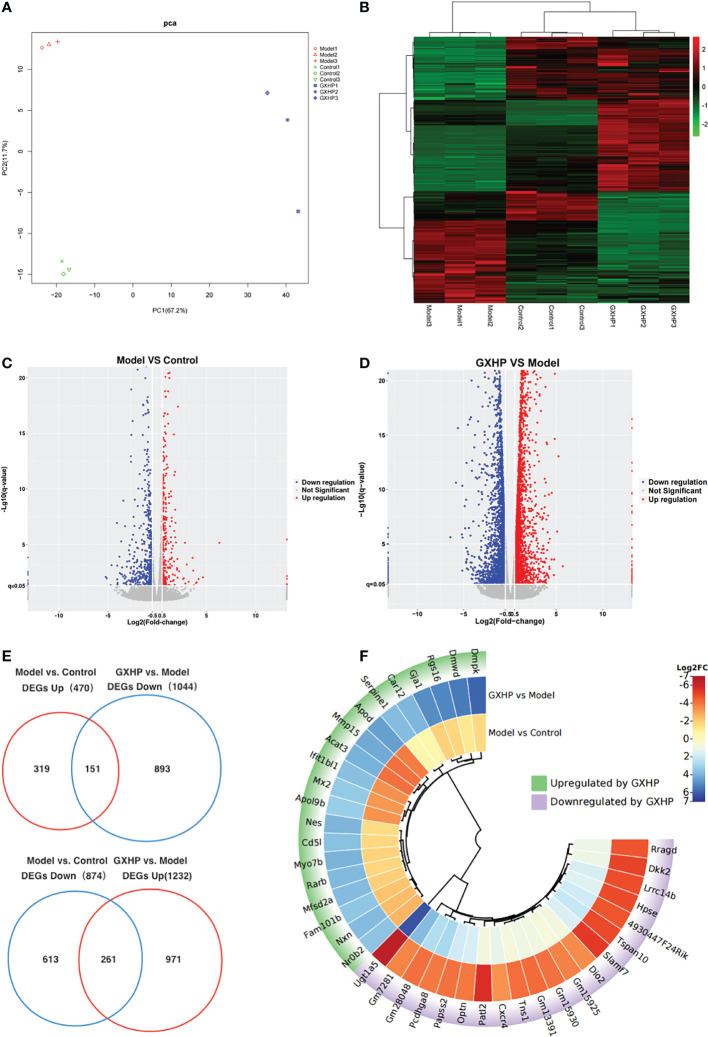
Effects of GXHP on RNA expression in foam cells. **(A)** PCA was performed to identify the clustering profiles of the differentially expressed genes (DEGs) among the three groups. The red clustered samples from model group (Models 1–3), the green clustered samples from control group (Controls 1–3), and the blue clustered samples from GXHP group (GXHP1–3). **(B)** Heatmap showing the overview of DEGs among the three groups. **(C)** Volcano plot showing DEGs between model and control groups. **(D)** Volcano plot showing DEGs between GXHP and Model groups. **(E)** Venn diagram showing the differentially downregulated and upregulated genes induced by ox-LDL and GXHP. **(F)** Circle heatmap showing the DEGs including the top 20 upregulated and downregulated genes in the model versus the control groups.

In this study, transcriptomic sequencing was performed to observe the DEGs among the three groups. Principle component analysis (PCA) and a heatmap showed that the three groups were clearly separated (**Figures 4A, B**). Compared with the control group, volcano plots showed 1,344 DEGs including 874 downregulated and 470 upregulated genes in the model group (**Figure 4C**). Moreover, there were 2,276 DEGs in the GXHP group compared with the model group (1,232 genes were upregulated and 1,044 were downregulated following GXHP treatment) (**Figure 4D**). Of the DEGs regulated by ox-LDL and GXHP, 412 DEGs affected the expression in opposite directions. Specifically, 151 DEGs that were upregulated by ox-LDL were downregulated by GXHP treatment, whereas 261 downregulated DEGs induced by ox-LDL were upregulated by GXHP treatment (**Figure 4E**). Moreover, the top 20 DEGs including upregulated and downregulated genes induced by GXHP treatment are shown in the circle heatmap (**Figure 4F**). These data suggest that GXHP treatment could restore gene expression changes induced by ox-LDL in foam cells.

### 3.5 Correlation between DNA methylation in promoter region and gene expression in foam cells treated with GXHP

**Figure 5:**
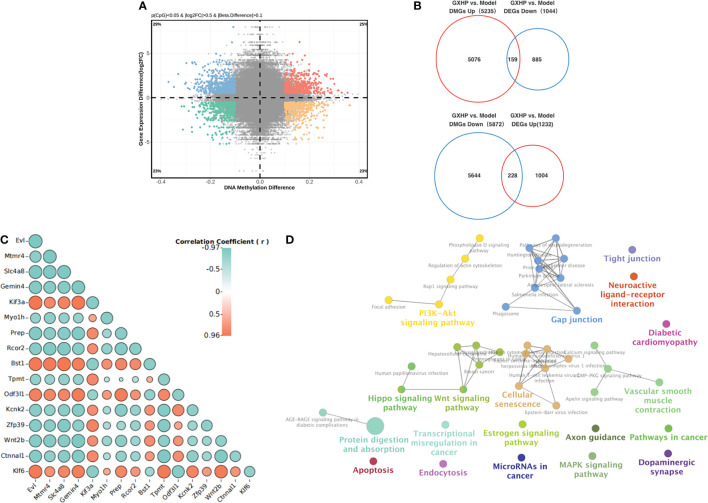
Integrative analysis of DNA methylation and RNA expression. **(A)** A scatter plot of mean methylation difference versus gene expression difference (log2FC). Each point represents a CpG-gene pair. **(B)** Venn diagrams summarizing the intersection for differentially hypermethylated genes and DEGs with upregulated or downregulated and differentially hypomethylated genes. **(C)** Correlation analysis of gene expression and DNA methylation was performed using Pearson correlation analysis. **(D)** KEGG pathways for the differentially hypermethylated genes with downregulated RNA expression and hypomethylated genes with upregulated RNA expression induced by GXHP.

Hypermethylation in the promoter region is associated with low expression of the genes. In contrast, hypomethylation is correlated with high expression of the corresponding gene. Here, we observed the opposite relationship between DEGs and DMGs in the cells from the model and GXHP groups. Compared with the model group, 387 DEGs/DMGs were identified in the GXHP group (**Figure 5A**). Specifically, there were 228 hypomethylated and 159 hypermethylated genes with upregulated and downregulated expressions, respectively (**Figure 5B**). Correlation analysis revealed the top 20 genes, which included Klf6, Evl, Mtmr4, and Slc4a8 (**Figure 5C**). Moreover, KEGG enrichment analysis indicated that these genes were closely related to the PI3K-Akt, Rap1, and Hippo signaling pathways (**Figure 5D**).

### 3.6 GXHP inhibited the PI3K-Akt signaling pathway in foam cells

**Figure 6:**
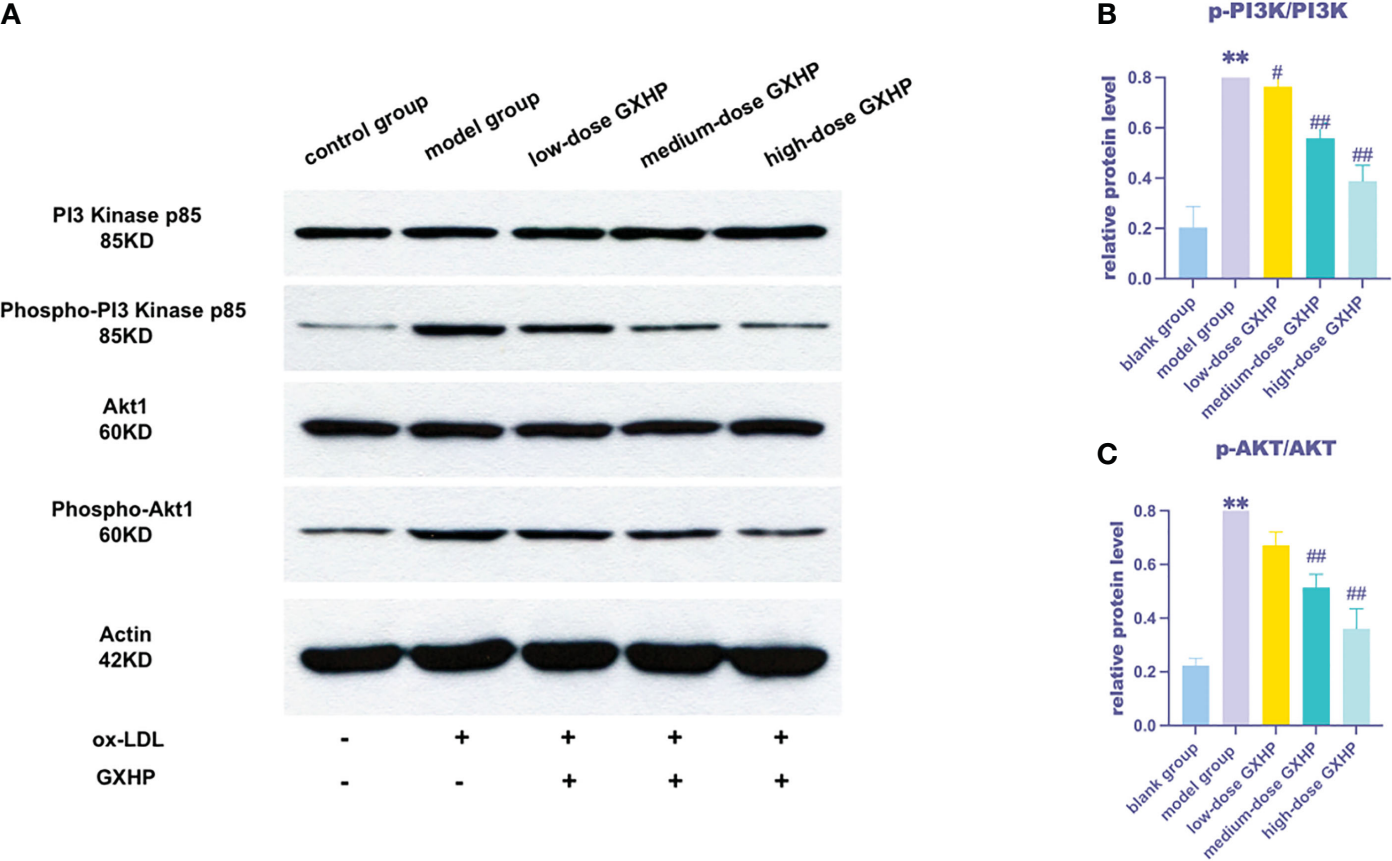
Effects of GXHP on protein expressions of PI3K-AKT in foam cells. **(A)** RAW 264.7 cells were treated with ox-LDL or GXHP (0, 0.2, 0.6, and 1.8 g/L) for 48 h, and western blotting was performed to determine the protein levels of PI3K, p-PI3K, AKT1, and p-AKT1. Gray values of **(B)** p-PI3K/PI3K and **(C)** p-AKT/AKT are shown. Data are presented as the mean ± SD of three independent experiments. **P < 0.01 compared with the control group; #*P* < 0.05 compared with the model group; ##*P* < 0.01 compared with the model group.

Based on the results of the correlation analysis, the PI3K-Akt signaling pathway was selected for validation. The expression of the key proteins p-PI3K, PI3K, p-AKT and AKT in foam cells was upregulated following ox-LDL treatment compared with that in the control group. However, GXHP treatment (1.8 and 0.6 g/L) decreased the ratios of p-PI3K/PI3K and p-AKT/AKT, indicating that GXHP can inhibit the PI3K-Akt signaling pathway in foam cells (**Figures 6A–C**).

## 4 Discussion

AS is the leading cause of morbidity and mortality worldwide, in which chronic inflammation coupled with elevated LDL cholesterol levels have been found to drive its development. Evidence suggests that Chinese herbal compounds in TCM are effective against AS (13, 14). This study reports the anti-AS effects of GXHP *via* DNA methylation changes *in vitro*. Our results suggest GXHP as a potential and effective agent for treating patients with AS.

Monocyte-derived cells absorb a proportion of ox-LDL and transform into foam cells (16–18); transformation of macrophages into lipid-loaded foam cells is an early event in the pathogenesis of AS. Furthermore, ox-LDL leads to the release of proinflammatory factors in foam cells, which accelerates the development of AS (19–21). Therefore, macrophages are attractive therapeutic targets for controlling the development of AS (22, 23). In the present study, foam cells were induced using ox-LDL at a concentration of 80 µg/mL for 48 h. Red lipid droplets and significant inflammatory cytokine upregulation were observed, which verified the successful establishment of a foam cell model. GXHP was then designed, and its anti-AS effects were tested in foam cells. A CCK-8 assay showed that foam cells could be safely treated with GXHP at 1.8 mg/mL, which was selected for subsequent experiments. Moreover, GC-MS and ELISA revealed that GXHP not only decreased the levels of TC and FC but also the expressions of IL-1β, TNF-α, and VCAM-1 compared with the model group. Taken together, our data suggest that GXHP provided a protective effect against AS.

Aberrant hypomethylation and hypermethylation play critical roles in the development of AS. For instance, aberrantly methylated genes including interferon-γ, intercellular adhesion molecule 1, nitric oxide synthase 3, 15-lipoxygenase, platelet-derived growth factor receptor alpha, and fatty acid desaturase 2 were found in AS. These hypomethylated or hypermethylated genes were closely associated with the inflammatory response, SMC proliferation, endothelial cell remodeling, and plaque development (24–28). Wang reported that the promoter region of miR-181b was hypermethylated in peripheral monocytes from patients with AS, and the hypermethylation of miR-181b could promote AS development (29). In the present study, we also found that many differentially hypermethylated and hypomethylated genes existed in foam cells induced by ox-LDL. KEGG enrichment analysis suggested that these abnormal DEGs were involved in many important pathways, such as the Rap1, Wnt, Hippo, and PI3K-Akt signaling pathways, which were closely associated with AS. Considering the unbalanced DNA methylation in AS, the development of drugs targeting aberrant hypermethylation and hypomethylation is vital for AS treatment in the future.

In this study, a genome-wide methylation analysis was applied to observe the change of DNA methylation in foam cells after treatment with GXHP, a TCM. GXHP treatment not only induced DNA hypermethylation, but also DNA hypomethylation. The number of aberrant hypomethylated and hypermethylated genes corrected by GXHP treatment was 3,213 (54.48%) and 2,685 (45.52%), respectively, which revealed that GXHP might be a novel methylation-based agent. These hypomethylated and hypermethylated genes induced by GXHP treatment were involved in the PI3K-Akt, Rap1, Hippo, and Wnt signaling pathways. Notably, most of those pathways play essential roles in the development of AS. Taken together, these results revealed that targeting aberrant hypermethylation and hypomethylation may be the main mechanism of action of GXHP in AS.

DNA methylation plays a crucial role in gene transcription. In the present study, we performed genome-wide expression analysis using RNA-seq. Consequently, GXHP treatment downregulated 151 DEGs that were upregulated by ox-LDL, and upregulate 261 DEGs that were downregulated by ox-LDL. The data demonstrated that GXHP treatment could correct the aberrant gene expression induced by ox-LDL. Hypomethylation in promoter regions could lead to over-expression of the genes, while hypermethylation suppresses expression. Our results showed that the mRNA expression of the 159 hypermethylated and 228 hypomethylated genes decreased and increased in the GXHP group compared with that in the model group, respectively. Pearson correlation analysis revealed the top DMGs and DEGs including Klf6, Evl, Mtmr4, and Slc4a8, which were considered potential and important treatment targets of GXHP. Previous studies have demonstrated that knockdown or inhibition of Klf6 expression could reduce macrophage filtration, reverse endothelial dysfunction, and lower inflammatory factor expression (30, 31).

Finally, KEGG enrichment analyses revealed that the DMGs and DEGs were involved in the PI3K-Akt, Rap1, Hippo and Wnt signaling pathways, among which PI3K-Akt signaling pathway ranked first. Previous studies showed that the PI3K-Akt signaling pathway is closely associated with inflammation in AS diseases (32, 33). Liu et al. found that inhibiting the expression of PI3K or AKT could decrease AS lesions and plaque areas and reduce the levels of IL-1β and NOD-like receptor protein 3 in ApoE^−/−^mice (34). Meng et al. (35) showed that morin hydrate, a naturally occurring bioflavonoid, could inhibit inflammation by inhibiting the PI3K/AKT1 signaling pathway in HUVECs treated with ox-LDL. Therefore, PI3K-Akt signaling pathway was chosen for the WB verification. WB showed that ox-LDL upregulated the protein levels of p-PI3K and p-AKT. More importantly, GXHP significantly downregulated the protein expression of p-PI3K and p-AKT in foam cells, suggesting the PI3K-Akt signaling pathway as an effective target for GXHP. However, *in vivo* experiments were not conducted in this study. Therefore, experiments with animal models are required to validate our findings in the future.

## 5 Conclusions

We demonstrated that GXHP showed anti-AS effects on foam cells. MC-seq combined with RNA-seq revealed that GXHP appeared to reverse gene expression changes *via* regulating aberrant hypermethylation and hypomethylation, thereby decreasing the levels of proteins involved in the PI3K-Akt signaling pathway in foam cells. Thus, GXHP may be a novel methylation-based agent. Clinical trials are needed to determine whether the protective effects of GXHP translate to better response in patients with AS in the future.

## Data availability statement

The data presented in the study are deposited in the Sequence Read Archive repository (https://dataview.ncbi.nlm.nih.gov/object). The accession numbers are SRR21846884, SRR21846885, SRR21846886, SRR21846887, SRR21846888, SRR21846883, SRR21846882, SRR21846881, SRR21846889, SRR21849088, SRR21849087, SRR21849095, SRR21849089, SRR21849090, SRR21849091, SRR21849092, SRR21849093 and SRR21849094.

## Author contributions

ZJ, YZ, YW, and HW contributed to the study design, practical work, and manuscript writing. JM, AW, FX, and QZ participated in the research design and manuscript revision. All authors contributed to data analysis and drafting or revising the article, and agree to be accountable for all aspects of the work. All authors contributed to the article and approved the submitted version.
